# Improving Access and Reducing the Burden on Public Ophthalmology: Collaborative Telehealth Models Between Ophthalmology and Optometry in Australia

**DOI:** 10.1007/s44402-026-00115-2

**Published:** 2026-06-11

**Authors:** Jingyi Chen, Khyber Alam, Nour Barakat, Stephen E. Bartnik, Sharon A. Bentley, Jocelyn J. Drinkwater, Allison M. McKendrick, Eileen Janet Richardson, Sandra C. Thompson, Kerry Woods, Angus W. Turner

**Affiliations:** 1 Department of Optometry and Vision Sciences, School of Health and Clinical Sciences, The University of Western Australia, Crawley, Western Australia Australia; 2 Northeastern State University Oklahoma College of Optometry, Tahlequah, Oklahoma USA; 3 Fremantle Hospital Ophthalmology Department, Fremantle, Western Australia Australia; 4 Lions Outback Vision, Broome, Western Australia Australia; 5 Herbert Wertheim School of Optometry and Vision Science, University of California Berkeley, Berkeley, California USA; 6 Centre for Ophthalmology and Visual Science, The University of Western Australia, Nedlands, Western Australia Australia; 7 Lions Eye Institute, Nedlands, Western Australia Australia; 8 Western Australian Centre for Rural Health, The University of Western Australia, Geraldton, Western Australia Australia

**Keywords:** Collaborative, Ophthalmology, Optometry, Telehealth

## Abstract

**Purpose:**

Collaborative care between optometry and ophthalmology has demonstrated the potential to improve timely access to care. This study examines three settings where real-time optometry-facilitated telehealth was used to expedite specialist eye care in rural and remote Western Australia. Referrals to ophthalmology were triaged to telehealth or face-to-face services. For telehealth, optometrists performed a comprehensive in-person assessment, then facilitated video-consultation with an ophthalmologist during the same attendance for collaborative decision making.

**Methods:**

In 2023, retrospective chart review was undertaken for ophthalmology services in towns more than 1500 km from the capital city. Optometrists performed comprehensive in-person assessments and facilitated telehealth in three settings: hospital, community clinic and visiting outreach. Attendance rates were compared between collaborative telehealth and face-to-face ophthalmology. Follow up outcomes and diagnoses for telehealth consultations were reported.

**Results:**

A total of 1876 non-surgical ophthalmology episodes of care were delivered in the 12-month period, of which 1044 (55.7%) were delivered by optometry using telehealth. Of those managed by telehealth, only 83 episodes of care required a subsequent face-to-face ophthalmology consultation. The hospital setting provided the greatest proportion (76.4%) of telehealth. Adjusted logistic regression showed the odds of attendance were 3.6 (95% CI: 2.6–5.0) times higher for telehealth appointments than face-to-face (*p* < 0.001). Surgical rates of outreach ophthalmology were high (30.0 to 74.1% of activity). Common diagnoses in telehealth included cataract, pterygium for direct surgical booking and chronic conditions (glaucoma, diabetic retinopathy) for instituting appropriate management.

**Conclusions:**

Collaborative telehealth with optometry improves access to ophthalmology services in rural Australia and should be considered in metropolitan settings and other countries.

Key points

- Collaborative telehealth between a local optometrist and remote ophthalmologist via videoconferencing resulted in high attendance rates and management of complex eye conditions without wait times or travel to see a specialist face-to-face.
- The workforce was used effectively, where optometrists managed chronic eye conditions and visiting ophthalmologists conducted high rates of surgery.
- Integrating collaborative telehealth into eye care frameworks shows potential to improve global eye health systems by supporting earlier intervention and enabling more efficient use of limited resources.

## Background

Eye health is a critical component of public health, and there has been increasing acknowledgement of the integral role that vision plays in advancing the United Nations Sustainable Development Goals [1]. Equitable access to eye care has received attention in recent years, with the World Health Organization launching several initiatives aimed at improving integrated people-centred approaches to eye care [2]. In Australia, there is an overall shortage of ophthalmologists and the workforce is unevenly distributed; only 16% are employed in the public sector, and many rural areas face persistent shortages due to workforce maldistribution [3]. These factors can contribute towards disproportionate delays in accessing public specialist ophthalmic care for people living in rural areas [4]. Adjunct modalities of care, such as telehealth, can play a critical part in overcoming geographic barriers and have an important role in improving timeliness of specialist outpatient services [5]. Telehealth has gained global momentum over recent years, expedited by the need to adapt during COVID-19 [6].

In Australia, optometrists outnumber ophthalmologists five-fold, are more geographically dispersed [7] and can assess, diagnose and manage a range of ocular conditions. Collaborative telehealth, whereby optometrists perform an in-person assessment and facilitate synchronous video-conferencing with an ophthalmologist, has been shown to improve access to ophthalmology services [8–12]. Although collaborative telehealth has been used in rural Western Australia by Lions Outback Vision since 2011, the COVID-19 pandemic catalysed the evolution of existing models [13]. Concurrently in 2020, Lions Outback Vision established a permanent eye clinic based in Broome, Western Australia. This service provided monthly ophthalmology visits to remote locations across the Pilbara and Kimberley regions and introduced a full-time on-demand statewide telehealth service for ophthalmology. While early models of telehealth relied on optometrists initiating a telehealth request solely for their own patients, a newer model evolved in 2020, where all public ophthalmology referrals were triaged to telehealth preferentially if clinically appropriate. Optometrists played a frontline role in managing ophthalmology referrals from general medical practitioners, emergency departments and other health professionals. This model allowed visiting ophthalmologists to focus on complex cases requiring face-to-face care, maximising the efficiency of in-person resources. The clinical team is supported by Aboriginal Liaison Officers and non-clinical staff who help support access to services for rural residents and Aboriginal and Torres Strait Islander Peoples through coordination, health promotion and education.

Despite the growing body of literature on telehealth involving optometry and ophthalmology, models are often disease-specific asynchronous models [14, 15] or are referred specifically for a telehealth service [9]. There is limited research exploring the role of optometrists in managing wait lists and referrals to ophthalmology directly using telehealth. Emerging models demonstrate the potential to streamline care and shorten wait times by allowing local optometrists to manage appropriate cases or facilitate direct surgical bookings via videoconferencing, reducing the ‘wait for wait’. Additionally, patients benefit from care delivered in familiar settings, minimising the need to travel and potentially enhancing continuity of care [16]. Wait times for cataract surgery in rural Australia are approximately twice as long as those in metropolitan regions [17]. Furthermore, wait time reports fail to account for the ‘invisible wait time’ or ‘wait for wait’ from initial referral to the time the patient is officially wait listed for surgery [18]. The personal implications of longer wait times for cataract surgery include loss of independence, loss of driver’s licence and increased risk of falls [18], with broader implications for health systems and the economy.

This retrospective study examines the evolving role of optometrists within three distinct rural Western Australian settings. In two locations, optometrists manage ophthalmology referrals: a regional hospital setting with an employed optometrist onsite and a community-based private optometry practice. The third setting is a remote Aboriginal outreach setting. The locations range from 1500 to 3000 km from the tertiary centre in the capital city and have monthly visiting ophthalmologist services for 1 to 2-day visits. This study aims to offer insight into the effectiveness and scalability of collaborative telehealth models in addressing specialist workforce shortages and improving access to eye care.

## Methods

### Telehealth model and clinical pathway

**Fig. 1:**
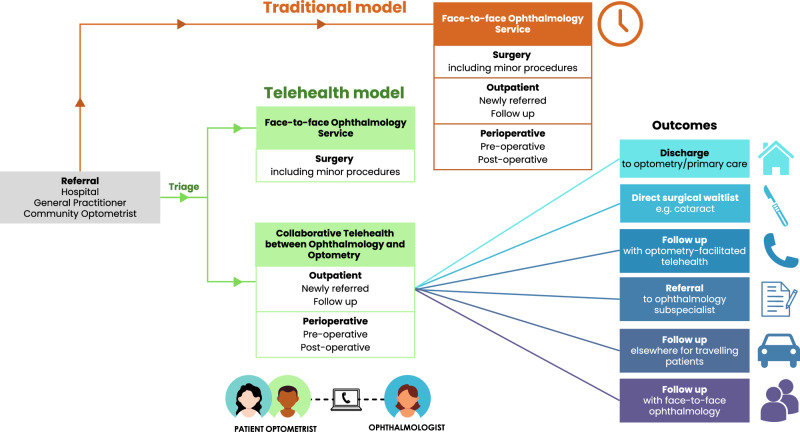
Pathway showing traditional model for ophthalmology services compared with collaborative telehealth model between ophthalmology and optometry. The traditional model is shown at the top in orange and the telehealth model is below in green. Follow up outcomes for the telehealth model are also presented.

The workflow for the telehealth clinical pathway, shown in Fig. 1, aimed to reduce duplication of clinical expertise between optometry and ophthalmology. All referrals to ophthalmology were triaged by registrars or optometrists; the referrals that required face-to-face specialist care, e.g., minor surgical procedures, were booked directly to visiting ophthalmology for in-person treatment. All other referrals, including undifferentiated cases, were triaged to telehealth. The types of appointments that could be seen using telehealth included outpatient and peri-operative (Fig. 1), which allowed increased allocation of limited visiting specialist time towards surgical cases.

Following triage, the optometrist performed an in-person comprehensive ocular assessment and then facilitated a synchronous telehealth consult during the same attendance with an on-call ophthalmologist. For telehealth, a video-link was established with the ophthalmologist, and three-way consultation between the patient, optometrist and ophthalmologist ensued. Shared electronic medical records, including remote viewing of ocular imaging between optometry and ophthalmology streamlined clinical entries and supported clinical decisions. The diagnostic devices available at the optometrist included slit lamp, tonometer, biometer, perimeter, digital retinal camera and optical coherence tomography. Remote viewing of images was enabled through proprietary web-based data management software or screen-sharing.

### Study design

The three settings presented in this study are summarised in Table 1. A retrospective 12-month audit of all ophthalmology medical records from all three locations was conducted. All scheduled ophthalmology appointments between 1 January 2023 and 31 December 2023, inclusive, were included, which encompassed outpatient and surgical services. The Modified Monash Model rural classification system was provided for each location, with locations in the current study being either Modified Monash (MM) 6 (remote communities) or MM 7 (very remote communities) [19].

**Table 1:** Health service characteristics.

	Service 1	Service 2	Service 3
**Setting**	Hospital optometry	Community optometry	Outreach optometry
**Modified Monash (MM) Model rural classification** [19]	MM 6	MM 6	MM 7
**Population (2021 Census)** [40]	4515	22,199	3222
**Description of service**	Employed optometrist in a public hospital. All patients referred to ophthalmology outpatients are seen by the optometrist in-person, and the patient and optometrist engage in telehealth with the ophthalmologist.	Community optometry private practice. The optometrist actively manages visiting ophthalmology referrals and triages suitable cases to scheduled telehealth clinics, during which the optometrist performs an in-person assessment and then engages in telehealth with the ophthalmologist.	Visiting optometrist provides outreach services for remote communities and does not triage ophthalmology referrals to telehealth.
**Approximate availability of face-to-face ophthalmology services**	13 days per year	13 days per year	13 days per year
**Telehealth use**	Patients are seen in regular telehealth clinics.	Referrals to ophthalmology are triaged to scheduled telehealth clinics. On-call telehealth is used for emergency cases.	No scheduled telehealth or triage. Opportunistic on-call telehealth used during outreach services.
**In-person collaboration between optometrists and visiting ophthalmologists**	The optometrist assists with visiting ophthalmology services.	The optometrist assists with visiting ophthalmology services.	The visiting optometrist is not present at the same time as the visiting ophthalmologist.
**Diagnostic equipment**	Slit lamp, tonometer, biometer, perimeter, digital retinal camera, optical coherence tomography.	Slit lamp, tonometer, biometer, perimeter, digital retinal camera, optical coherence tomography.	Slit lamp, tonometer, digital retinal camera.

The aim of this study was to answer:

i. How does telehealth influence the waiting list for visiting ophthalmology outpatient services? (Number of face-to-face appointments obviated, time from referral to first attendance and surgical case rate)
ii. What are the follow up outcomes of collaborative telehealth between optometry and ophthalmology?
iii. How does attendance rate compare between face-to-face and telehealth models of care for public ophthalmology?
iv. What eye conditions are managed via collaborative telehealth?

The time from referral to the first attendance was calculated for all newly referred outpatient episodes of care, excluding those where electronic referrals were unable to be accessed. Surgical case rate (SCR) was defined as the proportion of face-to-face appointments delivered by ophthalmologist consultants that were minor or major surgical procedures [20], which was used to assess the effective utilisation of the specialist-specific expertise during outreach visits. The SCR excluded face-to-face services delivered by Ophthalmology Registrars. Attendance rate was calculated by the proportion of appointments that were attended outside of scheduled appointments. Diagnoses were mapped to the International Classification of Diseases 11th Revision (ICD-11) using the ICD coding tool. For telehealth services, primary diagnoses were recorded. Follow up management outcomes of attended telehealth consultations were coded into six categories, as seen in Fig. 1: (i) Discharged; (ii) Waitlisted for surgery; (iii) Telehealth review; (iv) Face-to-face review; (v) Referred to subspecialist or metropolitan transfer or (vi) Follow up elsewhere for patients who were travelling and required follow-up with their usual provider interstate or overseas.

### Ethics

This study adhered to the tenets of the Declaration of Helsinki and was approved by the Western Australia Aboriginal Health Ethics Committee (HREC1371) with reciprocal approval from The University of Western Australia Human Research Ethics Committee (2024/ET000456). A waiver of consent was provided by the Western Australia Aboriginal Health Ethics Committee (HREC1371).

### Participants and data collection

Records where the patient was ≥ 18 years of age at the time of the appointment were included. Deidentified data were extracted into an abstraction form [21] and manually coded by the first author (JC), an optometrist who was not involved in the care delivery. Regular meetings were held between research team members to clarify any discrepancies in data coding and interpretation.

### Data analysis

Descriptive and inferential analyses were undertaken using IBM SPSS Statistics for Windows, version 29 (ibm.com). Demographics for individual patients at their first presentation to the service were summarised using descriptive statistics. Four patients had two new referrals within the study period; therefore, only the first attendance was included in analysis of time from referral to assessment. Data are given as number (percentage), mean ± standard deviation for normally distributed data and median [inter-quartile range] where data were not normally distributed. Bivariate analyses were by chi-square for categorical variables, independent *t*-test for normally distributed continuous variables or Mann–Whitney *U* test where data were not normally distributed. Multivariate logistic regression was used to determine whether telehealth, age and gender influenced the odds of attendance.

## Results

A total of 3131 public ophthalmology episodes of care for 1212 patients were scheduled between 1 January 2023 and 31 December 2023 across all three sites. This included attended and non-attended scheduled outpatient services (i.e., telehealth or face-to-face), peri-operative consultations (pre-operative and post-operative) and surgery. There were 1529 face-to-face services delivered by ophthalmology consultants and 88 delivered by registrars; the former were used in SCR calculations. The average age of patients was 56 ± 15 years, and 51.4% (*n* = 623) were male. There were 391 patients (32.3%) with diabetes and 535 (44.1%) identified as Aboriginal and/or Torres Strait Islander. A total of 2661 services were attended in-person (85.0%).

### Referral source

There were 578 newly referred attendances. Referrals were uploaded electronically for 470 attendances (81.3%), which accounted for 171 and 299 that were seen face-to-face and using telehealth, respectively. The most common referral source was optometry (*n* = 295, 62.8%), followed by hospital (*n* = 89, 18.9%) and general medical practitioner (*n* = 73, 15.5%). There were 13 referrals (2.8%) from private ophthalmology, nurses, registrars and support workers.

### Waiting list

In the 12-month period, there were 1309 attended outpatient episodes of care, of which 747 (57.1%) were delivered using collaborative telehealth and thus avoided a face-to-face ophthalmology outpatient appointment (Table 2). Service 1 (Hospital) had the highest proportion of telehealth appointments, followed by Service 2 (Community) and Service 3 (Outreach).

**Table 2:** Telehealth as a proportion of ophthalmology outpatient episodes of care delivered, and time from referral to first attendance for newly referred outpatient consultations.

All attended services (*n* = 2661)					
	Face-to-face		Telehealth		Total
**Outpatient services**	562 (42.9%)		747 (57.1%)		1309
**Peri-operative**	270 (47.6%)		297 (52.4%)		567
**Surgery**	785 (100%)		0 (0%)		785
**Total**	1617		1044		2661
**Face-to-face services delivered by ophthalmology consultants (*n*** = **1529)**					
	**Service**	**Service 1 Hospital**	**Service 2 Community**	**Service 3 Outreach**	**Total**
**Surgical case rate**	**Surgical procedures**	256 (47.3%)	371 (74.1%)	146 (30.0%)	785 (51.3%)
**Attended ophthalmology outpatient services (*n*** = **1309)**					
**Episodes of care**	**Face-to-face ophthalmology**	166 (23.6%)	199 (52.0%)	197 (88.7%)	562 (42.9%)
	**Collaborative telehealth between optometry and ophthalmology**	538 (76.4%)	184 (48.0%)	25 (11.3%)	747 (57.1%)
	**Total**	704	383	222	1309
**Time from referral to first attendance for newly referred outpatient episodes of care (n** = **470)**					
**Time days**	**Face-to-face**	6 [0–44]	21 [6–98]	33 [8–99]	22 [3–90]
	**Telehealth**	7 [0–36]	14 [2–43]	0 [0–0]	7 [0–34]

Data given as *n* (%) or median [interquartile range] unless otherwise specified.

Non-electronically uploaded referrals (*n* = 108, 18.7%) were excluded from the analysis looking at the time from referral to assessment; however, the services were otherwise included for analysis of attendance, diagnoses and outcomes. The median waiting time for telehealth assessments (Table 2) was 7 days, compared with 22 days for face-to-face appointments (*p* < 0.001).

### Attendance

Attendance rates for face-to-face and telehealth appointments were 79.4% and 95.3%, respectively. There was no significant difference in attendance between genders (*p* = 0.12). Increasing age was associated with a greater likelihood of attendance (*p* < 0.001). The odds of attendance were 3.6 (95% CI: 2.6–5.0) times higher for those who had a telehealth appointment than face-to-face after adjusting for age, community and Aboriginal status (*p* < 0.001).

### Outcomes

**Fig. 2:**
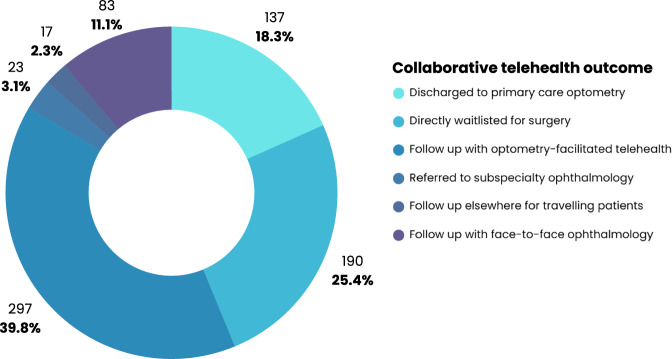
Follow up outcomes per outpatient episode of care delivered using collaborative telehealth. The outcomes are organised in a clockwise manner and the number of episodes of care and percentage are displayed alongside each outcome.

The follow up management outcomes for collaborative telehealth outpatient episodes of care (*n* = 747) can be seen in Fig. 2.

Of the 1044 telehealth appointments, there were 113 episodes of care (10.8%) where no diagnosis of ocular or systemic pathology was made, such as when screening for diabetic retinopathy was undertaken. The primary diagnoses for other telehealth appointments grouped by ICD-11 classification (Supplementary File 1) are summarised in Table 3.

**Table 3:** Primary diagnoses for attended telehealth episodes of care, sorted by frequency (*n* = 931).

Diagnosis			Frequency
Cataract			370
Glaucoma			89
*Primary open-angle glaucoma*		57	
*Ocular hypertension*		17	
*Glaucoma suspect*		15	
Pterygium			50
Diabetes			42
*Diabetic retinopathy*		28	
*Diabetic macular oedema*		14	
Keratitis, e.g., bacterial, punctate			38
Macular conditions e.g., cystoid macular oedema, epiretinal membrane, macular hole, central serous chorioretinopathy			26
Corneal foreign body			22
Conjunctivitis			21
*Adenoviral conjunctivitis*	14		
*Allergic conjunctivitis*	7		
Refractive error			21
Anterior uveitis			15
Benign neoplasm of eye or ocular adnexa			14
Age-related macular degeneration			12
Retinal breaks or detachments			12
Corneal abrasion			10
Dermatochalasis			10
Herpes simplex or zoster, e.g., keratitis, keratouveitis			9
Retinal vein occlusion			9
Keratoconjunctivitis sicca			8
Posterior capsular opacification			7
Posterior vitreous detachment			7
Chalazion			7
Superficial injury of eyelid or periocular area			7
Chemical burn of eye or ocular adnexa			6
Binocular vision, e.g., strabismus, amblyopia			5
Conjunctival or subconjunctival haemorrhage			5
Malignant neoplasm of eye or ocular adnexa			5
Recurrent erosion of cornea			5
**Other diagnoses**^**a**^			
Other - lid and adnexa			23
Other - cornea			21
Other - choroid and retina			15
Other - optic nerve			9
Other - conjunctiva			7
Other - lens			8
Other - unclassified			8
Other - sclera			4
Other - neurological			2
Other - vitreous			2

^a^Detailed diagnoses can be found in Supplementary files.

## Discussion

This study found that collaborative telehealth between optometry and ophthalmology substantially obviated the need for specialist face-to-face assessment. The odds of attendance were 3.6 times higher for telehealth compared with face-to-face appointments. The overall time between referral and attendance was shorter for telehealth compared with face-to-face; however, with Service 1 (Hospital) and Service 2 (Community), the embedded and full-time availability of the telehealth system increased availability of visiting face-to-face ophthalmology services. This may explain the shorter time for face-to-face appointments observed in Service 1 (Hospital).

Across all three rural locations, surgical case rates were higher compared to previously reported Australian ophthalmology outreach clinics [20]. Service 1 (Hospital) and Service 2 (Community) had higher surgical case rates than Service 3 (Outreach). Despite the telehealth use in Service 3, which expedites surgery, the lack of integrated collaborative care and telehealth triage processes may have limited the efficiency of the clinic. Conversely, Services 1 and 2 demonstrated improved efficiencies of the telehealth model, likely through avoiding duplication of clinical expertise by triaging specialist visits effectively.

This study highlighted the role of optometrists in outpatient management and in pre- and post-operative care [22]. Internationally, the role of optometrists facilitating telehealth has been explored previously in the literature, though nearly all of these examples were asynchronous models [5, 14, 15, 23, 24]. In Australia, the Community Eye Care (C-EYE-C) model uses asynchronous telehealth for diabetes [25] and glaucoma [26] management. In the United Kingdom, similar shared care models utilising asynchronous telehealth exist for glaucoma [27, 28]. In addition to exploring the work of optometrists within telehealth systems, recent years have seen a shift towards increased community care in efforts to reduce demand on an overburdened public hospital system [29].

Other telehealth studies have found that shortened wait times were linked with improved attendance rates [30]. Attendance rates for traditional ophthalmology outreach services in Western Australia at Aboriginal Medical Services have been reported to range from 37% to 61% in the past [31]. It has been suggested that attendance improves with telehealth as it overcomes barriers such as transportation [32]. The availability of same-day telehealth also reduces barriers for patients who have travelled a long distance to see the referring provider [8]. A large proportion of the patients in this study identified as Aboriginal and/or Torres Strait Islander (44.1%) compared with the national average of 3.8% [33], which highlights the importance of culturally safe care beyond clinical issues in eye care delivery. Factors such as cultural support, care coordination, health promotion and education likely contributed to the high attendance rates observed in this study [34, 35]. High telehealth attendance might also be attributable to familiarity with a culturally responsive local provider [31, 36]. Furthermore, appropriate triage will influence the success of telehealth, which also depends on the content and quality of referrals [37, 38]. In the present investigation, the primary diagnoses for telehealth showed a proportion of referrals to ophthalmology that could be suitable for primary care optometry, such as screening (*n* = 113, 10.8%) and refractive error (*n* = 21, 2.0%), further emphasising the importance of appropriate referral.

This study demonstrates a long-term sustainable model of eye care access, harnessing the efficiencies of collaborative telehealth in a workforce poor and geographically isolated region. Its application in three different clinical eye care settings have the potential to be translated to other contexts. With the ageing population and predicted rise in chronic conditions, efficient and innovative models of healthcare delivery, such as through effective telehealth, will be necessary [39]. Future research should explore the scalability of this model for adoption in other jurisdictions, including metropolitan settings. While distance is less of a barrier in urban areas, barriers to timely public ophthalmology care remain, such as wait times over 1 year for cataract assessment in Australia [38]. Economic evaluation is needed to establish the cost-effectiveness of this telehealth model and inform resource allocation.

### Limitations

The aim of this study was to explore outcomes of telehealth; therefore, patient and clinician perspectives were not explored. A limitation of retrospective analysis is that coding was subject to uncertainty in some cases. To avoid potential misrepresentation of results, uncertain cases were identified in the results section and excluded from analysis rather than making assumptions. Another limitation is that the longstanding nature of the service (since 2011) means that prospective pre- and post-implementation comparison within the same setting was not possible. Therefore, variations in demographics and service delivery models were described within the case study to provide context.

Patients were triaged to telehealth or face‑to‑face care based on clinical details provided in the referral. This potential selection bias may have contributed to the observed differences in attendance rates and wait times. Collaborative telehealth would be valuable for underserved communities or any system where the current needs are unable to keep up with demand. However, the findings in the current study may not be generalisable to less resourced or urban settings. Therefore, implementation in different settings would require careful consideration of contextual factors.

### Policy implications

This research presents evidence to support the role that optometry can play in managing the public ophthalmology burden in Australia. The outcomes demonstrate how telehealth has been used in remote hospitals, private community-based optometry and visiting outreach settings for Aboriginal populations. Lions Outback Vision is able to deliver this service with no cost barriers for patients due to public State health block funding for ophthalmology. The hospital optometrist is a salaried position also supported by block funding, whereas the community optometrist relies on Federal funding through Medicare, Australia’s universal public health-insurance scheme. Optometrists employed in public hospitals are uncommon in rural Australia [7], yet the findings of this study support their value and the benefits of expanding funded positions. In the broader Australian context, there are no formal incentives for optometrists or ophthalmologists to use telehealth and no quantifiable targets set by professional regulators, State or Federal governments. Incentives may be helpful to promote uptake of the telehealth model. This study may inform future trial design for asynchronous models of telehealth, potentially improving cost-effectiveness, and to advocate for funding support.

## Conclusion

This study demonstrated the role of optometry in obviating specialist face-to-face services in rural Australian settings. Telehealth enables a reduction in the duplication of clinical expertise and increased access to care. Collaborative telehealth was associated with shorter times from referral to appointment and improved attendance compared with face-to-face clinics. The follow-up management outcomes of telehealth showed that most telehealth appointments did not require a face-to-face ophthalmology follow up. Collaborative telehealth between optometry and ophthalmology has potential to be used in other regional and metropolitan settings around Australia and internationally, to increase access to timely eye care.

## Supplementary information

Supplementary File_Telehealth models

## Data Availability

The datasets used and analysed during the current study are not shared publicly for privacy and ethical reasons. They may be available from the corresponding author on reasonable request.
