# Supplementary material for: Improving Access and Reducing the Burden on Public Ophthalmology: Collaborative Telehealth Models Between Ophthalmology and Optometry in Australia
Source: Ophthalmic Physiol Opt. 2026 Jun 11;46(4):959–66. doi: 10.1007/s44402-026-00115-2 (PMC13395816; doi:10.1007/s44402-026-00115-2)
Supplement: Supplementary file 1 — Supplementary File_Telehealth models [file 44402_2026_115_MOESM1_ESM.docx]

**Supplementary Table: International Classification of Diseases (ICD)-11 diagnoses for collaborative telehealth between ophthalmology and optometry (n=931)**

| **Grouping** | **ICD code** | **ICD-11 Diagnosis** | **Total** |
| --- | --- | --- | --- |
| Cataract | 9B10.Z | Cataract, unspecified | 370 |
| Primary open-angle glaucoma | 9C61.0Z | Primary open-angle glaucoma, unspecified | 57 |
| Pterygium | 9A61.1 | Pterygium | 50 |
| Refractive error | 9D00.Z | Disorders of refraction, unspecified | 21 |
| Corneal foreign body | ND70.0 | Foreign body in cornea | 18 |
| Keratitis, e.g. bacterial | 9A7Z | Disorders of the cornea, unspecified | 18 |
| Ocular hypertension | 9C61.01 | Ocular hypertension | 17 |
| Diabetic retinopathy | 9B71.00&XS0T | Moderate nonproliferative diabetic retinopathy | 16 |
| Keratitis, e.g. bacterial | 9A71&XN74M | Bacterial keratitis | 16 |
| Adenoviral conjunctivitis | 1D84.0 | Conjunctivitis due to adenovirus | 14 |
| Glaucoma suspect | 9C60 | Glaucoma suspect | 13 |
| Diabetic macular oedema | 9B71.02 | Diabetic macular oedema | 10 |
| Corneal abrasion | NA06.4&XA4C02 | Corneal abrasion injury | 10 |
| Dermatochalasis | 9A03.5 | Dermatochalasis of eyelid | 10 |
| Anterior uveitis | 9A96.Z | Anterior uveitis, unspecified | 10 |
| Macular conditions e.g. CMO, ERM, hole, CSCR, drusen | 9B7Y | Other specified disorders of the retina | 8 |
| Keratoconjunctivitis sicca | 9A79 | Keratoconjunctivitis sicca | 8 |
| Age-related macular degeneration | 9B75.0Z | Age-related macular degeneration, unspecified | 7 |
| Allergic conjunctivitis | 9A60.02 | Allergic conjunctivitis | 7 |
| Chalazion | 9A02.0Z | Chalazion, unspecified | 7 |
| Retinal breaks or detachments | 9B73.Y | Other specified retinal detachments or breaks | 7 |
| Superficial injury of eyelid or periocular area | NA00.1Z | Superficial injury of eyelid or periocular area, unspecified | 7 |
| Posterior capsular opacification | 9B10.22 | After-cataract | 7 |
| Posterior vitreous detachment | 9B81 | Posterior vitreous detachment | 7 |
| Chemical burn of eye or ocular adnexa | NE00 | Burn of eye or ocular adnexa | 6 |
| Anterior uveitis | 9A96.Z | Anterior uveitis, unspecified | 5 |
| Benign neoplasm of eye or ocular adnexa | 2F36.Y&XA3RB1 | Papilloma of eyelid | 5 |
| Conjunctival or subconjunctival haemorrhage | 9A61.5 | Conjunctival or subconjunctival haemorrhage | 5 |
| Diabetic retinopathy | 9B71.01 | Proliferative diabetic retinopathy | 5 |
| Herpes simplex or zoster, e.g. keratitis, keratouveitis | 1F00.1Y | Herpes simplex anterior uveitis | 5 |
| Recurrent erosion of cornea | 9A78.8 | Recurrent erosion of cornea | 5 |
| Retinal venous occlusions | 9B74.1 | Retinal venous occlusions | 5 |
| Binocular vision, e.g. strabismus, amblyopia | 9C8Z | Strabismus or ocular motility disorders, unspecified | 4 |
| Corneal foreign body | ND70.0 | Foreign body in cornea | 4 |
| Diabetic retinopathy | 9B71.00&XS5W | Mild nonproliferative diabetic retinopathy | 4 |
| Macular conditions e.g. CMO, ERM, hole, CSCR, drusen | 9B75.2 | Central serous chorioretinopathy | 4 |
| Macular conditions e.g. CMO, ERM, hole, CSCR, drusen | 9B78.3Y | Other specified degeneration of macula or posterior pole | 4 |
| Macular conditions e.g. CMO, ERM, hole, CSCR, drusen | 9B75.1 | Non-traumatic macular hole | 4 |
| Other - cornea | 9A78.20 | Bullous keratopathy | 4 |
| Other - cornea | 9A78.50 | Keratoconus | 4 |
| Retinal venous occlusions | 9B74.1 | Retinal venous occlusions | 4 |
| Other - lens | 9B11.1 | Dislocation of lens | 4 |
| Keratitis, e.g. bacterial | 9A7Y | Other specified disorders of the cornea | 4 |
| Other - lid and adnexa | 9A03.1Z | Entropion of eyelid, unspecified | 4 |
| Other - lid and adnexa | 9A03.2Z | Ectropion of eyelid, unspecified | 4 |
| Age-related macular degeneration | 9B75.04 | Neovascular late-stage age-related macular degeneration | 3 |
| Benign neoplasm of eye or ocular adnexa | 9A06.Y | Other specified disorders of eyelid | 3 |
| Diabetic macular oedema | 9B71.02 | Diabetic macular oedema | 3 |
| Diabetic retinopathy | 9B71.00&XS25 | Severe nonproliferative diabetic retinopathy | 3 |
| Herpes simplex or zoster, e.g. keratitis, keratouveitis | 1F00.10 | Herpes simplex keratitis | 3 |
| Macular conditions e.g. CMO, ERM, hole, CSCR, drusen | 9B75.Z | Macular disorders, unspecified | 3 |
| Macular conditions e.g. CMO, ERM, hole, CSCR, drusen | 9B75.Z | Macular disorders, unspecified | 3 |
| Malignant neoplasm of eye or ocular adnexa | 2C32.Z | Basal cell carcinoma of skin, unspecified | 3 |
| Other - conjunctiva | 9A60.Y&XN74M | Bacterial Conjunctivitis | 3 |
| Other - cornea | 9A76 | Corneal ulcer | 3 |
| Other - lens | 9B11.0 | Aphakia | 3 |
| Other - lid and adnexa | 9A01.0 | Preseptal cellulitis | 3 |
| Other - optic nerve | 9C40.A0 | Papilloedema | 3 |
| Other - sclera | 9B50 | Episcleritis | 3 |
| Other - optic nerve | 9C40.AZ | Optic disc swelling, unspecified | 3 |
| Retinal breaks or detachments | 9B73.4 | Retinal breaks without detachment | 3 |
| Age-related macular degeneration | 9B78.3Z | Degeneration of macula or posterior pole, unspecified | 2 |
| Benign neoplasm of eye or ocular adnexa | 2F36.Z | Benign neoplasm of eye or ocular adnexa, unspecified | 2 |
| Glaucoma suspect | 9C61.10 | Primary angle closure suspect or anatomical narrow angle | 2 |
| Malignant neoplasm of eye or ocular adnexa | 2D00.Z | Malignant neoplasm of conjunctiva, unspecified | 2 |
| Other - choroid and retina | 2F36.0 | Benign neoplasm of choroid | 2 |
| Other - cornea | 9A70.Y | Other specified hereditary corneal dystrophies | 2 |
| Other - cornea | 9A77.Y | Other specified corneal scars or opacities | 2 |
| Other - lid and adnexa | NA06.02 | Oedema of eyelid | 2 |
| Other - neurological | 8B8Y | Other specified disorders of cranial nerves | 2 |
| Other - UNCLASSIFIED e.g. endophthalmitis, transient vision loss | NA02.2Z | Orbital fracture, unspecified | 2 |
| Other - vitreous | 9B83 | Vitreous haemorrhage | 2 |
| Retinal breaks or detachments | 9B73.Y | Other specified retinal detachments or breaks | 2 |
| Other - choroid and retina | 9B71.1 | Hypertensive retinopathy | 2 |
| Other - choroid and retina | 9B78.3Y | Other specified degeneration of macula or posterior pole | 2 |
| Other - lid and adnexa | 9A02.1 | Posterior blepharitis | 2 |
| Other - UNCLASSIFIED e.g. endophthalmitis, transient vision loss | LD2D.1Z | Neurofibromatosis, unspecified | 2 |
| Benign neoplasm of eye or ocular adnexa | 2F36.Z | Benign neoplasm of eye or ocular adnexa, unspecified | 1 |
| Benign neoplasm of eye or ocular adnexa | 2F36.Z | Benign neoplasm of eye or ocular adnexa, unspecified | 1 |
| Benign neoplasm of eye or ocular adnexa | 2F36.1 | Benign neoplasm of iris | 1 |
| Benign neoplasm of eye or ocular adnexa | EK70.3 | Hidrocystoma | 1 |
| Binocular vision, e.g. strabismus, amblyopia | 9D46 | Impairment of binocular functions | 1 |
| Diabetic macular oedema | 9B71.02 | Diabetic macular oedema | 1 |
| Herpes simplex or zoster, e.g. keratitis, keratouveitis | 1E91.1 | Ophthalmic zoster | 1 |
| Other - choroid and retina | 9B6Z | Disorders of the choroid, unspecified | 1 |
| Other - choroid and retina | 2D05.0 | Melanoma of choroid | 1 |
| Other - choroid and retina | 9B78.8 | Retinal ischaemia | 1 |
| Other - choroid and retina | 9B78.4 | Peripheral retinal degeneration | 1 |
| Other - choroid and retina | 9B62 | Chorioretinal scars | 1 |
| Other - choroid and retina | NA06.6Z | Traumatic injuries of the retina, unspecified | 1 |
| Other - choroid and retina | 9B65.1 | Infectious posterior uveitis | 1 |
| Other - choroid and retina | 9B78.5 | Retinal haemorrhage | 1 |
| Other - conjunctiva | 9A61.4Y | Other specified conjunctival vascular disorders | 1 |
| Other - conjunctiva | NA06.4&XA8PS3 | Injury of conjunctiva | 1 |
| Other - conjunctiva | 9A6Y | Other specified disorders of conjunctiva | 1 |
| Other - conjunctiva | 9A61.0 | Pingueculae | 1 |
| Other - cornea | 9A78.2Z | Corneal oedema, unspecified | 1 |
| Other - cornea | 9A70.Y | Other specified hereditary corneal dystrophies | 1 |
| Other - cornea | 9A73 | Exposure keratitis | 1 |
| Other - cornea | 9A70.0 | Endothelial corneal dystrophy | 1 |
| Other - cornea | 9A7Y | Other specified disorders of the cornea | 1 |
| Other - cornea | NA06.8D | Ocular laceration without prolapse or loss of intraocular tissue, unilateral | 1 |
| Other - lens | NA06.8C&XA13U9 | Retained intraocular nonmagnetic foreign body, bilateral [Crystalline lens] | 1 |
| Other - lid and adnexa | 8A02.00 | Benign essential blepharospasm | 1 |
| Other - lid and adnexa | 9A11.1 | Canaliculitis | 1 |
| Other - lid and adnexa | NA06.01 | Haematoma of eyelid | 1 |
| Other - lid and adnexa | 9A06.Y | Other specified disorders of eyelid | 1 |
| Other - lid and adnexa | 9A03.0Z | Blepharoptosis, unspecified | 1 |
| Other - lid and adnexa | LC10 | Dermal melanocytosis | 1 |
| Other - lid and adnexa | 9A21.0 | Orbital cellulitis | 1 |
| Other - lid and adnexa | 9A11.4 | Punctal stenosis | 1 |
| Other - optic nerve | LA13.72 | Congenitally elevated optic disc | 1 |
| Other - optic nerve | 9C40.1Z | Optic neuritis, unspecified | 1 |
| Other - optic nerve | 9C40.Y | Other specified disorder of the optic nerve | 1 |
| Other - sclera | 9B51 | Scleritis | 1 |
| Other - UNCLASSIFIED e.g. endophthalmitis, transient vision loss | 9C21.Z | Endophthalmitis, unspecified | 1 |
| Other - UNCLASSIFIED e.g. endophthalmitis, transient vision loss | 2F37.0 | Non-secreting pituitary adenoma | 1 |
| Other - UNCLASSIFIED e.g. endophthalmitis, transient vision loss | 9D51 | Transient visual loss | 1 |
| Other - choroid and retina | 9B78.9 | Retinal atrophy | 1 |
| Other - UNCLASSIFIED e.g. endophthalmitis, transient vision loss | 8D60.Y | Other specified increased intracranial pressure | 1 |
